# An Account of Experiments and a Post-Mortem Examination; Illustrating the Physiology of the Excito-Motor System of Nerves

**Published:** 1853-08

**Authors:** S. B. Hunt

**Affiliations:** Buffalo, N. Y.


*An account of Experiments and a post-mortem Examination; Illustrating the Physiology of the Excito-motor System of Nerves.* By S. B. Hunt, M. D., Buffalo, N. Y.

On the 12th of July ult., Dr. Bailey, of this city, requested Dr. Bowen and myself to visit an acephalous infant, born at 11 P. M., the night previous. We found the child alive, but apparently moribund. With the consent of the mother, we removed it to Dr. Bailey's office.

The case presented the following features:—From the superior part of the frontal sinuses, the calvarium sloped directly backward; being covered with an unusually thick scalp. At the point of the anterior fontanelle, there was a deep fissure in the integuments, extending transversely, and adherent to the skull at its base. From the posterior fontanelle, protruded a tumor inverted in a membrane reflected from the cerebral cavity. This tumor resembled precisely, and undoubtedly was, the cerebellum inverted in the dura mater. Otherwise the child was very well formed.

It breathed irregularly—respirations from five to ten per minute, and suspirious in character. Pulse, at the carotids, 70. Surface cold. When undisturbed it is perfectly motionless; but if its extremities are pinched, it withdraws them. Reflex and ganglionic life are present; but we could obtain no evidence of perception or volition. When the settee on which it lay was rudely jarred, it would start. It uttered no cry, except when the tumor was dragged upon, it gave the sound peculiar to lesion or disturbance of the medulla oblongata. It swallowed water, and sucked vigorously when the finger was placed in its mouth. The eyes moved, and the pupil contracted when the lids were opened.

It will be seen from this description, that all muscular movements dependent upon the spinal axis were complete, as were the ganglionic actions. Sucking, deglutition, respiration, prehension, iritic contractility, and the circulation were all there; and yet the child was, (so far as any living soul, mind, perception, or volition were concerned,) to all intents and purposes, dead. This will be verified by the post-mortem, which took place on the evening of the 13th. The child had then been dead a few hours, having been returned to its mother for proper care as long as it should continue to breathe.

*Autopsia.* Scalp very much thickened, and reflected upon itself. Carried an incision from ear to ear, and everted the anterior flap. The posterior flap was split down, and the tumor isolated at its neck. The bones were unusually firmly ossified, and their location was so abnormal as to confuse the dissection. The calvarium was so depressed that the surface of the calvarial dura mater was in contact with, and in some places adherent to, the base of the skull. On removing the calvarium, the sella turcica (considerably distorted) and the fossæ of the base of the skull came immediately into view. There was an entire absence of the cerebrum. The cerebellum lay (as before stated) on the outside of the cranium. At the posterior fontanelle this cerebellar tumor narrowed into an apology for a medulla oblongata, which projected directly downward into the spinal canal. From the sides of this pediole various nervous filaments originated, and passed out at the foramina and fissures of the skull. It was impossible to recognize them, but there could be seen no optic tract or thalami, however rudimentary. Probably the first and second pairs were not developed. The existence of the third, fourth, and first branches of the fifth pair was demonstrated by the experiments before death. Also the sympathetic, glosso-pharyngial, facial, and I believe from the symptoms present, all those cerebral nerves which, originating in the cerebullum or medulla oblongata, are distributed to muscles. There was no *pons varolii.*

This case is interesting inasmuch as it demonstrates that a cerebrum is not essential to organic and reflex life — this child having lived thirty-six hours without one. It also proves that muscular motions, even those apparently exhibiting judgment, such as the withdrawal of the foot from the pinch of the forceps, are not necessary volitional, or at any rate have no connection with any action of the brain. The reflex actions exhibited by this child furnished the same evidence which Dr. Dowler derived from his "Nilotic saurian" *(Anglice alligator)* experiments; but they do not to my mind, in any manner contradict the physiology of Marshall Hall; neither do they prove the existence of volition apart from cerebral action. For volition and perception must necessarily have the same locale in the nervous system, and though reflex motions may *appear* volitional, it is simple absurdity to suppose perception resident in any part but the cerebrum.

